# Impaired pre-competition wellbeing measures can negatively impact running performance in developmental youth female soccer players

**DOI:** 10.5114/biolsport.2024.129480

**Published:** 2023-07-21

**Authors:** Michael G. Sydney, Martin Wollin, Dale W. Chapman, Nick Ball, Jocelyn K. Mara

**Affiliations:** 1 University of Canberra Research Institute for Sport and Exercise (UCRISE), Canberra, Australia; 2 School of Science, Faculty of Science & Technology, University of Canberra, Australia; 3 Discipline of Sport and Exercise Science, Faculty of Health, University of Canberra, Australia; 4 School of Allied Health, Curtin University, Perth, Australia; 5 Performance Health Management, Canberra, Australia; 6 School of Health Sciences, La Trobe University, Melbourne, Australia

**Keywords:** Menstrual cycle, Stress, Fatigue, Lower-body muscle soreness, GPS, Self-reporting questionnaires

## Abstract

This study aimed to determine the association between pre-competition perceived player wellbeing measures and subsequent relative and peak running performance of developmental youth female soccer players (n = 15, age: 16 ± 1 years). Total distance (TD), high-speed (> 3.5 m/s) (HSRD) and very high-speed (> 5.3 m/s) running (VHSRD) were expressed using 1-, 2- and 5-minute epochs and relative (per minute) calculations. Fatigue, sleep quality, upper and lower-body muscle soreness, stress, and mood wellbeing measures were collected via a self-reported questionnaire (1–5 Likert scale). Menstrual cycle phase was collected via a calendar-based countback method. Results demonstrated that reductions in stress was associated with decreased relative and peak TD in all epochs (*p* = 0.008–0.040), relative and peak HSRD (*p* = 0.006–0.039) in 2- and 5-minute epochs as well as VHSRD in 2-minute epochs (*p* = 0.026). For example, a one-point reduction of ‘normal’ to ‘relaxed’ is associated with a decrease of 7 m/min in peak TD for 1-minute epochs. One-point increase in fatigue (e.g., ‘normal’ to ‘more tired than normal’) displayed a decrease of 7 m/min peak TD for 2-minute (*p* = 0.048) and 9 m/min for 5-minute (*p* = 0.007) rolling epochs. Likewise, one-point increase in lower-body muscle-soreness (e.g., ‘normal’ to ‘increase in soreness/tightness’) was associated with a reduction of 6 m/min peak VHSRD for 1-minute epochs (*p* = 0.034). Results suggest that perceived player wellbeing can influence running performance. However, the magnitude of the change in player wellbeing should be considered in a practical sense.

## INTRODUCTION

In team sports such as soccer, an integrated approach to athlete monitoring is essential when seeking to assess player wellbeing and promote optimal performance [1, 2]. Perceived player wellbeing questionnaires are commonly used in high-performance sport to provide a cost-effective, accurate and sensitive evaluation of athletes’ wellbeing [1, 2]. Typically formulated utilising 5-, 7- or 10-point Likert scales, these questionnaires monitor fluctuations in self-reported fatigue, sleep quality, muscle soreness, stress and mood [3–7]. Subjective wellbeing measures have demonstrated superior sensitivity and consistency compared to objective measurements such as biochemical markers when assessing responsiveness to acute and chronic loads [8]. The development and implementation of online athlete monitoring apps and wellbeing questionnaires have minimised the necessity for regular, onerous biochemical testing procedures to monitor players [2, 8]. In addition, these apps and questionnaires promote the integration of sex specific factors, such as menstrual cycle phase, into female athlete monitoring practices [9–11]. In turn, supporting coaches, practitioners and medical staff to develop sex specific, evidence-based approaches to maximise competition readiness in female athletes [9–11].

In combination with variables such as player wellbeing and menstrual cycle, metrics related to accumulative (e.g., total distance covered in a match) and relative (e.g., average distance covered per minute of match time) running performance provide valuable data to coaches and practitioners. In particular, increasing importance has been placed on the evaluation of peak running performance [12–15]. For example, Schimpchen et al., [12] reported that sequences of peak high-speed (> 5 m/s) intensity were evenly distributed across 15-minute fixed time periods (e.g., 0–15, 15–30, 30–45 etc.) during competition matches. However, the greatest number of peak intensity sequences for peak total distance, accelerations and decelerations, using 1-, 5- and 10-minute rolling epochs, occurred in the first 15-minutes of match-play [12]. Likewise, Mäkiniemi et al., [16] observed that elite senior female Finnish soccer players covered less total and high-speed (> 3.6 – 5.3 m/s) running distance in the last 15-minute period compared to the first 15-minute period of match-play. As such, the first 15-minute fixed time period provides a practical window of assessing the most demanding period of competition match-play [12, 16].

To date, investigations have reported that reductions in pre-training wellbeing negatively influenced running performance in elite male Australian Football (AFL) [6] and soccer players [3]. Further, Gaelic football [4] and female Lacrosse [17] athletes have reported that pre-training and pre-competition measures of sleep quality, sleep duration and muscle soreness were associated with subsequent changes to running performance in training and competition match-play. While these findings suggest that coaches and practitioners can utilise perceived player wellbeing measures as indicators of readiness to meet these demands, the data reported was limited to accumulative and relative running performance measures [3, 4, 6, 17]. Additionally, these results are not transferrable across sports or genders, and the relationship between pre-competition player wellbeing and subsequent running performance in developmental youth female soccer players remains unknown. Moreover, there is a current lack of investigations that consider menstrual cycle phase in their statistical modelling, which may be a confounding variable in female athlete research [10, 11, 18–20]. In turn, the exclusion of menstrual cycle phase hinders the translation of research to applied practice in female sporting environments [10, 11, 18–20]. It is important to connect changes in pre-competition perceived player wellbeing, accounting for menstrual cycle phase, to practical, significant changes in female soccer players’ running performance [10, 11, 18–20]. Thus, the purpose of this study was to determine the association between pre-competition perceived player wellbeing measures and subsequent relative and peak running performance of developmental youth female soccer players.

## MATERIALS AND METHODS

### Experimental approach to the problem

This study employed a longitudinal observational design. Data was collected on outfield developmental youth female soccer players (n = 15) during a single Australian National Premier League Women’s (NPLW) competition season. The NPLW was classified as a sub-elite competition and matches were played throughout a seven-month competition period (March – September). Perceived player wellbeing metrics were collected daily via an online wellbeing questionnaire using mobile devices. The running performance for each player during match-play were captured using a global positioning system (GPS). Players were categorised according to playing position in each match.

### Participants

Fifteen (n = 15) outfield academy youth female soccer players (age: 16 ± 1 yrs.; height 165 ± 6 cm; body mass 59 ± 7 kg; Yo-Yo Intermittent Running Test Level 2 (YYIR2) score; 640 ± 48 m) participated in this study. Participants were recruited from the same Australian W-League youth academy program and classified as Tier 2: developmental [21]. A typical training week consisted of 2–3 soccer specific on-field sessions, 1–2 competition matches, and 1–2 gym-based resistance training sessions. Ethical approval was granted by the University of Canberra Human Research Ethics Committee (project number: 1990). All players and parents or guardians were informed about the requirements, risks, and benefits of participation in this study prior to providing their informed written consent.

### Procedures Player Wellbeing

Players completed an online wellbeing questionnaire via smart phone devices each day between 8:00 AM and 10:00 AM. Players were instructed not to discuss their responses with other players, coaches, staff, parents, or guardians. The questionnaire has been previously utilised and adapted from other studies [7, 22], developed according to recommendations for implementing customised wellbeing questionnaires in athletes [23]. Each variable was measured on a five-point Likert scale (Table 1), and consisted of variables related to fatigue, sleep quality, upper-body and lower-body muscle soreness, stress, and mood [7, 22, 23].

**TABLE 1:** Perceived Player Wellbeing Questionnaire, adapted from Wellman et al., [7] and Abbott et al., [22].

Category	Player Response				

	1	2	3	4	5
Fatigue	Always Tired	More Tired Than Normal	Normal	Fresh	Very Fresh

Sleep Quality	Insomnia	Restless Sleep	Difficulty Falling Asleep	Good	Very Restful

Upper-Body Muscle Soreness	Very Sore	Increase in Soreness/Tightness	Normal	Feeling Good	Feeling Great

Lower-Body Muscle Soreness	Very Sore	Increase in Soreness/Tightness	Normal	Feeling Good	Feeling Great

Stress Levels	Highly Stressed	Feeling Stressed	Normal	Relaxed	Very Relaxed

Mood	Highly Annoyed / Irritable / Down	Snappiness at Team Mates / Family	Less Interested than usual	Generally Good Mood	Very Positive Mood

Wellbeing scores from three, two and one day prior to competition as well as morning of competition were collated for analysis to account for the day-to-day fluctuations in player wellbeing as a response to lingering effects. Where players had failed to complete their daily perceived wellbeing questionnaire in one of the days leading to the day of competition, missing data were imputed using an exponential weighted moving average (EWMA) using that player’s wellbeing scores from the two preceding and two succeeding days [24]. The use of an EWMA ensured that more weight was given to more recent wellbeing values [24]. A final four-day EWMA score was calculated for each wellbeing variable and included the wellbeing scores of the three preceding days leading to the day of competition, as well as the day of competition.

### Menstrual Cycle Phase

The players menstrual cycle phase on a given day was categorised into two phases, follicular and luteal which was determined using a calendar-based counting method [10, 25]. In addition to their daily wellbeing questions, players were asked “*Did you have your period today?*” [10, 25]. A change in responses from “no” to “yes” established the onset of menses and the start of the follicular phase [10, 25]. Furthermore, players were asked “*What was the date of your last period (first day of menstruation)?*”. This allowed the luteal phase to be calculated using a retrospective calendar countback method [10, 25]. Where possible, the days in which players did not complete their daily wellbeing, menstrual cycle phase was calculated from completed questionnaires using the preceding and succeeding days [10, 25]. Where players had not yet started menarche or were experiencing amenorrhoea, defined as the absence of menstrual period > 3 months to ≥ 6 months [11], menstrual cycle phase was categorised as ‘irregular’.

### Running Performance

Each player completed an average of 11 competition matches (range = 6–22), resulting in a total of 165 individual match files for analysis. Playing positions were categorised as central defenders (CD, n = 24 total match files), external defenders (ED, n = 39 total match files), midfielders (MD, n = 45 total match files), external attackers (EA, n = 47 total match files) and central attackers (CA, n = 10 total match files). To account for participants featuring in different playing positions throughout the competition season, players positions were categorised for each individual match accordingly. Data were trimmed so that only the first 15-minutes of on-field playing time was included in the analysis to minimise the influence of any changes in peak running performance ascribed to accumulative match fatigue, score line differences, half-time intervals and other in-game contextual influences. No tactical substitutions took place during this time. Players who were substituted during the first 15-minutes due to injury were omitted from the analysis. During all data collection competition matches, a 4-3-3 formation was used.

Players running performance was collected using 15 Hz global positioning system (GPS) devices (SPI HPU, GPSports, Canberra, Australia). Devices were worn between the scapulae in a fitted garment to limit device movement. Each player was allocated the same GPS device for the duration of the data collection to minimise the effect of interunit error. Each device was turned on 30-minutes prior to the match warm up, to ensure satellite connectivity. Between 4 to 12 satellites were available for connectivity and signal transmission during match-play, satisfying the criteria for ideal positional detection [26]. The horizontal dilution of precision (HDOP) was not reported by the proprietary software (Team AMS, Canberra, Australia). Captured metrics included total distance (TD), high-speed running distance (HSRD), defined as distance covered at > 3.5 m/s, and very high-speed running distance (VHSRD), defined as distance covered at > 5.3 m/s [14]. These thresholds have previously been used for elite youth female soccer players [14]. The inter-unit reliability of the GPS devices (expressed as a coefficient of variation) has been reported as 1.4% for total distance, 7.8% for distance at speeds between 2.0–5.9 m/s, and 4.8% for distance covered at speeds > 5.9 m/s [27].

Following each match, data were downloaded using Team AMS software (GPSports, Canberra, Australia). The TD, HSRD and VHSRD were expressed as relative (i.e., TD/min, HSRD/min, and VHSRD/min) and peak values (i.e., peak TD, peak HSRD and peak VHSRD) for the first 15-minutes. To calculate the peak demands, GPS data were split into 30-second intervals and 1-, 2- and 5-minute rolling sums were calculated. Previous research has reported that fixed epochs underestimate total (7–10%) and high-speed (12–25%) running distance (defined as > 5.5 m/s) in elite senior male soccer players [13] and as such, rolling epochs have been utilised when quantifying peak running performance in elite youth female soccer players [14]. Peak running performance was calculated as the maximum TD, HSRD and VHSRD achieved in each 1-, 2- and 5-minute rolling window. To allow comparison between the different epoch lengths, TD, HSRD and VHSRD were expressed as per minute values relative to epoch length time.

### Statistical analysis

Statistical analyses were conducted using R version 4.2.3 [28] and RStudio version 2023.03.0+386 [29]. Separate Linear Mixed Models (LMM) for each epoch length (1-, 2-, 5-minutes) and relative calculations were conducted using the *lmer* function from the *lme4* package [30] to determine the association between the EWMA well-being variables (fatigue, sleep quality, upper-body muscle soreness, lower-body muscle soreness, stress levels and mood scores) (fixed factors) and the peak and relative TD, HSRD and VHSRD (dependant variables). For each LMM, menstrual cycle phase was included as a random factor. In addition, the player position and the unique player identification number were included as nested random factors. Random effects have been reported in the results section as *position:athlete*_RE_ for the variance attributed to the nested random factors, and *menstrual cycle*_RE_ for the variance attributed to the menstrual cycle phase. A Type II Wald F test was conducted using the *Anova* function from the *car* package [31] to determine the significance (alpha = 0.05) of main effects. The assumption of normality was determined upon visual inspection of histograms and Q-Q plots of the residuals. Multicollinearity was inspected for each model prior to analysis using the *vif* function from the *car* package [31]. All fixed factors were found to have *vif* scores less than 5.0, suggesting no evidence of multicollinearity. The assumptions of homoscedasticity and linearity were confirmed upon visual inspection of plots of the fitted values against the residuals [32].

## RESULTS

**FIG. 1:**
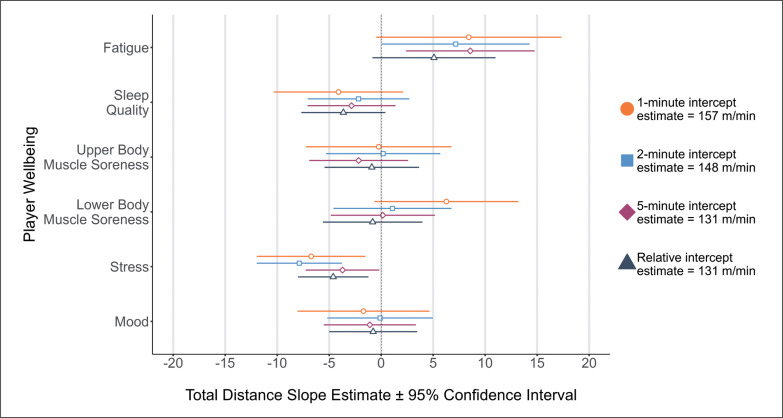
Total Distance (TD) for 1-, 2-, 5-minute rolling epoch lengths and relative calculations according to changes in player wellbeing.

Main effects were identified between stress and relative TD/min (*p* = 0.008, *position:athlete*_RE_ = *55.24, menstrual cycle*_RE_ *= 0.00*), peak TD in 1- (*p* = 0.011, *position:athlete*_RE_ = *46.62, menstrual cycle*_RE_ *= 0.00*), 2- (*p* < 0.001, *position:athlete*_RE_ = *0.00, menstrual cycle*_RE_ *= 0.00*) and 5-minute (*p* = 0.040, *position:athlete*_RE_ = *0.52, menstrual cycle*_RE_ *= 0.00*) rolling epochs, with reduction in stress (e.g., ‘normal’ to ‘relaxed’) associated with lower running performance (Figure 1). Main effects for fatigue were found for peak TD in 2- (*p* = 0.048, *position:athlete*_RE_ = *0.00, menstrual cycle*_RE_ *= 0.00*) and 5-minute (*p* = 0.007, *position:athlete*_RE_ = *0.52, menstrual cycle*_RE_ *= 0.00*) rolling epochs, with increased fatigue (e.g., ‘normal’ to ‘more tired than normal’) associated with reduced running performance (Figure 1).

**FIG. 2:**
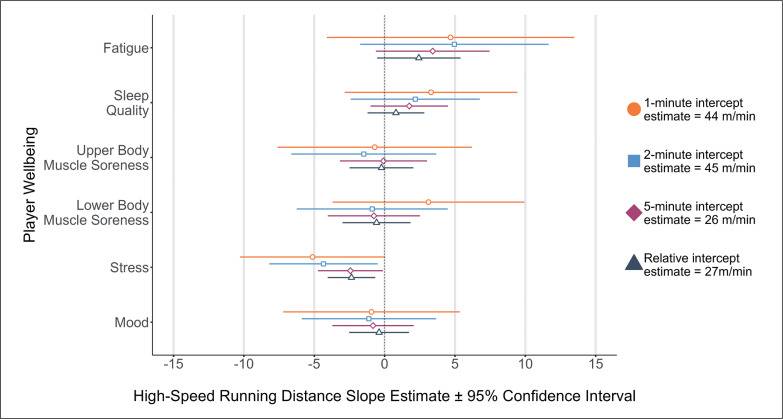
High-Speed Running Distance for 1-, 2-, 5-minute rolling epoch lengths and relative calculations according to changes in player wellbeing.

Main effects were identified for stress and relative HSRD/min (*p* = 0.006, *position:athlete*_RE_ = *0.01, menstrual cycle*_RE_ *= 0.00*) and peak HSRD in 2- (*p* = 0.027, *position:athlete*_RE_ = *50.10, menstrual cycle*_RE_ *= 3.43*) and 5-minute (*p* = 0.039, *position:athlete*_RE_ = *3.31, menstrual cycle*_RE_ *= 0.57*) rolling epochs, respectively, where reduced stress (e.g., ‘feeling stressed’ to ‘normal’) was associated with lower high-speed running performance (Figure 2).

**FIG. 3:**
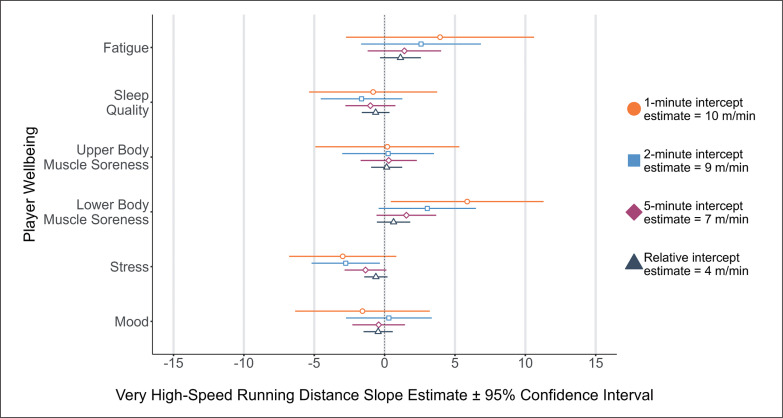
Very High-Speed Running Distance for 1-, 2-, 5-minute rolling epoch lengths and relative calculations according to changes in player wellbeing.

Main effects were identified for lower-body muscle soreness and peak VHSRD in 1-minute (*p* = 0.034, *position:athlete*_RE_ = *11.07, menstrual cycle*_RE_ *= 0.00*) rolling epochs, indicating increased lower-body muscle soreness (e.g., ‘normal’ to ‘increase in soreness/tightness’) was associated with lower VHSRD (Figure 3). Main effects were also identified for stress and VHSRD in 2-minute (*p* = 0.026, *position:athlete*_RE_ = *0.00, menstrual cycle*_RE_ *= 0.00*) rolling epochs, indicating that reduced stress was associated with lower VHSRD (Figure 3). All other perceived player wellbeing metrics were non-significant across peak TD, HSRD and VHSRD in 1-, 2- and 5-minute rolling epochs as well as relative calculations (*p* = 0.051 -0.967).

## DISCUSSION

The purpose of this study was to determine the association between pre-competition perceived player wellbeing measures and subsequent relative and peak running performance of developmental youth female soccer players. The findings of this investigation suggest that reductions in stress (e.g., ‘feeling stressed’ to ‘normal’) were associated with a decrease in relative and peak TD, relative and peak HSRD as well as decreased peak VHSRD. Reported increases of fatigue (e.g., ‘normal’ to ‘more tired than normal’) were associated with decreased peak TD. Increased lower-body muscle soreness (e.g., ‘normal’ to ‘increase in soreness/tightness’) was also found to be associated with decreased peak very high-speed running. These findings provide evidence to the concept that pre-competition perceived player well-being may influence future running performance in early competition match-play in developmental youth female soccer players. Furthermore, a strength in the design of this investigation was the inclusion of menstrual cycle phase as a random factor. The methodology employed in this study provides a simple example of how non-invasive approaches can be employed to increase the quantity and quality of research in female athletes.

Several studies have cited perceptual measures of wellbeing influencing subsequent running performance in AFL [6], field hockey [33], lacrosse [17], and soccer [3] with the use of individual perceptual wellbeing measures (e.g., stress) being more appropriate to inform an expected running output from players rather than a sum of wellbeing measures in Gaelic football [4]. The results of this study agree with previous findings, demonstrating that stress appears to be associated with changes in running performance. For example, Figure 1 shows that a one-point change in a player’s perceived stress levels (e.g., ‘feeling stressed’ to ‘normal’) is associated with a decrease of 7 m/min in peak TD for 1-minute rolling epochs. Whilst a decrease of 7 m/min for peak TD may not signal a practically meaningful change in running performance, the magnitude in the change of player wellbeing should be considered in a practical sense. For example, a three-point change in a player’s perceived stress levels (e.g., ‘highly stressed’ to ‘relaxed’) would result in a decrease of 21 m/min in peak TD for 1-minute rolling epochs. Likewise, a one-point change in perceived stress was associated with reductions of 4 m/min at high-speed and 3 m/min at very high-speed during a 2-minute epoch. Therefore, a three-point change in a player’s perceived stress levels would result in a decrease of 12 m/min at high-speed and 9 m/min at very high-speed. As such, it appears that the magnitude of change in ‘stress’ responses prior to competition determines whether the subsequent change in high and very high-speed running performance is trivial (i.e., -4 or -3 m/min), or meaningful (i.e., -12 or -9 m/min) in 2-minute rolling epochs. A player’s anticipation to perform in competition has been reported to influence salivary cortisol concentration levels, reflective of increases in cognitive and somatic anxiety preceding competition [34]. In this regard, the increased peak running performance associated with reported perceived states of ‘feeling stressed’ and ‘highly stressed’ could be reflective of arousal to perform as opposed to negative state of mental wellbeing. Additionally, these findings could be potentially attributed to the level of competition and participant classification in this investigation [21, 34]. National and regional level players have reported increased cognitive anxiety responses prior to competition when compared to international level athletes [34]. Further research is required to determine whether the impact of stress differs between international, national, and regional youth female soccer players.

A player’s ability to accelerate, run at near maximal velocity and change direction at high-speed is critical in soccer and commonly underpins vital match situations such as scoring goals [35, 36]. Previous research has indicated that changes in pre-training ratings of fatigue and composite wellness scores can limit subsequent running performance, which in turn may lead to a detriment in the ability to successfully perform actions at high-speed in crucial match situations [3, 6, 17, 35, 36]. This study reports that increased fatigue were associated with reductions in peak TD in 2- and 5-minute rolling epochs. Developmental youth female soccer players displayed an average decrease of 7 m/min and 9 m/min for 2- and 5-minute rolling epochs, respectively, when reporting a one-point change (e.g., ‘normal’ to ‘more tired than normal’) in fatigue. Consequentially, a three-point change would constitute a decrease of 21 m/min for 2- and 27 m/min for 5-minute rolling epochs. However, considering that there was no reported association between fatigue and relative high-speed and very high-speed running or peak high-speed and very high-speed running, the results imply that the change in peak TD associated with fatigue is reflective of player running performance at < 3.5 m/s (i.e., low speed running). It appears alterations of running performance associated with pre-competition fatigue, whilst statistically significant, provide little to no indication of substantial impairment regarding player running performance during critical, high-speed scenarios such as those observed prior to goal-scoring moments [35, 36].

Our results suggest that measures of lower-body muscle soreness are associated with changes in subsequent running performance during competition. For example, a one-point change in player lower-body muscle-soreness (e.g., ‘normal’ to ‘increase in soreness/tightness’) elicits a decrease in peak very high-speed running of 6 m/min. Practically, this means a three-point change in a player’s lower-body muscle-soreness would result in a decrease of 18 m/min in a 1-minute epoch. A decrease of 6 m/min over the course of 1 minute of match-play would unlikely hinder players in critical match situations. However, a decrease of 18 m/min at very high-speed across a 1-minute epoch would constitute a significant, meaningful change in running performance. Our results further highlight the need to consider the magnitude of change in perceived player wellbeing metrics prior to competition and its association with player’s subsequent running performance. However, there was no association between lower-body muscle soreness and peak very high-speed running in 2-, 5-minutes rolling epochs and relative measures. Coaches and practitioners should therefore remain cautious when associating reported pre-competition perceived lower-body muscle soreness and subsequent running performance.

Although perceived player wellbeing measures from the 72-hour period following competition were not investigated in this study, it is important to consider these findings in relation to the practicality of utilising perceived player wellbeing throughout an entire training microcycle. For example, Evans et al., [37] reported that stress, sleep, and total wellness measurements five days prior (MD-5) to competition were indicative of the total number of accelerations and decelerations in the following match but not TD, HSRD (> 4.1 m/s) or VHSRD (> 5.2 m/s) in under-18 elite youth male soccer players. Other studies have found that players reported significant reductions in wellbeing during the 72-hours following competition [3, 5]. Malone et al., [3] reported that in elite senior (age: 25.3 ± 3.1) male soccer players reduction in wellbeing negatively impacted running performance in training environments. Notably, the lowest wellbeing Z-scores (Z-score of -2) were reported the day following competition (MD+1) with a steady increase throughout the microcycle with the greatest player wellbeing being reported on match day [3]. These findings suggest that player wellbeing observed in the 72-hour window following competition appear to have an influence on subsequent competition running performance of players [3, 37]. Therefore, the results observed in this study may be reflective of the exclusion of perceived player wellbeing in the 72-hours following competition and therefore not accounting for the greatest decrement in perceived player wellbeing during a typical training microcycle.

Whilst these findings suggest that pre-competition perceived player wellbeing measures are associated with subsequent player running outputs, the results of this study should be interpreted in accordance with several limitations. Firstly, menstrual cycle phase was only separated into follicular and luteal phases. Future investigations should seek to utilise the proposed definitions of 1) early follicular, 2) late follicular, 30) ovulatory and 4) midluteal in future investigations [11]. Furthermore, the use of hormonal contraception by players should be included as an additional covariate in future studies [11]. Finally, the influence of ‘survey fatigue’ has been reported when undertaking athlete wellbeing research and as such, results should be interpreted with this in mind [38].

## CONCLUSIONS

This study is the first to investigate the association between precompetition perceived player wellbeing, accounting for menstrual cycle phase, and the relative and peak running outputs of developmental youth female soccer players. Stress, fatigue, and lower-body muscle soreness appear to be the key metrics that are associated with subsequent changes in running performance in developmental youth female soccer players. Practically, coaches and practitioners could use changes in perceived player wellbeing metrics as indicators to proactively initiate targeted communication and gain more information regarding an athlete’s state prior to competition. However, the scale of change must be considered, as small one-point changes of perceived player wellbeing may not provide a meaningful change in running performance. Alternatively, a three-point change will likely impact subsequent running performance and in this regard may be used as the foundation for a face-to-face conversation in the lead up to competition fixtures. Nonetheless, such considerations should be made in alignment with normal athlete response patterns and context of personal circumstances. Finally, the study design and data reported in this investigation provides the basis for coaches and practitioners to promote and continue the inclusion of menstrual cycle tracking into athlete monitoring practices to further understand the multifaceted nature of performance in female athletes.
